# Emergence of wheat blast in Bangladesh was caused by a South American lineage of *Magnaporthe oryzae*

**DOI:** 10.1186/s12915-016-0309-7

**Published:** 2016-10-03

**Authors:** M. Tofazzal Islam, Daniel Croll, Pierre Gladieux, Darren M. Soanes, Antoine Persoons, Pallab Bhattacharjee, Md. Shaid Hossain, Dipali Rani Gupta, Md. Mahbubur Rahman, M. Golam Mahboob, Nicola Cook, Moin U. Salam, Musrat Zahan Surovy, Vanessa Bueno Sancho, João Leodato Nunes Maciel, Antonio NhaniJúnior, Vanina Lilián Castroagudín, Juliana T. de Assis Reges, Paulo Cezar Ceresini, Sebastien Ravel, Ronny Kellner, Elisabeth Fournier, Didier Tharreau, Marc-Henri Lebrun, Bruce A. McDonald, Timothy Stitt, Daniel Swan, Nicholas J. Talbot, Diane G. O. Saunders, Joe Win, Sophien Kamoun

**Affiliations:** 1 Department of Biotechnology, Bangabandhu Sheikh Mujibur Rahman Agricultural University, Gazipur, 1706 Bangladesh; 2 Plant Pathology, Institute of Integrative Biology, ETH Zurich, 8092 Zurich, Switzerland; 3 INRA, UMR 385 Biologie et génétique des interactions plantes-pathogènes BGPI, Montpellier, France; 4 College of Life and Environmental Sciences, University of Exeter, Exeter, EX4 4QD UK; 5 Earlham Institute, Norwich Research Park, Norwich, NR4 7UH UK; 6 Argo-Environmental Remote Sensing and Modeling Lab, Bangladesh Agricultural Research Institute, Joydebpur 1701, Gazipur, Bangladesh; 7 Directorate of Grains Industry, Department of Agriculture and Food Western Australia (DAFWA), 3 Baron-Hay Court, South Perth, WA 6151 Australia; 8 Brazilian Agricultural Research Enterprise - EMBRAPA Wheat/Trigo, Passo Fundo, Rio Grande do Sul Brazil; 9 Department of Crop Protection, Rural Engineering, and Soil Science, University of São Paulo State - UNESP, IlhaSolteira Campus, São Paulo, Brazil; 10 CIRAD, UMR 385 Biologie et génétique des interactions plantes-pathogènes BGPI, Montpellier, France; 11 The Sainsbury Laboratory, Norwich Research Park, Norwich, NR4 7UH UK; 12 Max Planck Institute for Plant Breeding Research, Carl-von-Linné-Weg 10, Cologne, 50829 Germany; 13 INRA, UMR 1290 Biologie et Gestion des Risques en agriculture BIOGER, Thiverval-Grignon, France; 14 John Innes Centre, Norwich Research Park, Norwich, NR4 7UH UK

**Keywords:** Field pathogenomics, Wheat blast, Phylogenomic analysis, *Eleusine indica*, *Oryza sativa*

## Abstract

**Background:**

In February 2016, a new fungal disease was spotted in wheat fields across eight districts in Bangladesh. The epidemic spread to an estimated 15,000 hectares, about 16 % of the cultivated wheat area in Bangladesh, with yield losses reaching up to 100 %. Within weeks of the onset of the epidemic, we performed transcriptome sequencing of symptomatic leaf samples collected directly from Bangladeshi fields.

**Results:**

Reinoculation of seedlings with strains isolated from infected wheat grains showed wheat blast symptoms on leaves of wheat but not rice. Our phylogenomic and population genomic analyses revealed that the wheat blast outbreak in Bangladesh was most likely caused by a wheat-infecting South American lineage of the blast fungus *Magnaporthe oryzae*.

**Conclusion:**

Our findings suggest that genomic surveillance can be rapidly applied to monitor plant disease outbreaks and provide valuable information regarding the identity and origin of the infectious agent.

**Electronic supplementary material:**

The online version of this article (doi:10.1186/s12915-016-0309-7) contains supplementary material, which is available to authorized users.

## Background

Outbreaks caused by fungal diseases have increased in frequency and are a recurrent threat to global food security [1]. One example is blast, a fungal disease of rice, wheat, and other grasses, that can destroy enough food supply to sustain millions of people [1–3]. Until the 1980s, the blast disease was not known to affect wheat, a main staple crop critical to ensuring global food security. In 1985, the disease was first reported on wheat (*Triticum aestivum* L.) in Paraná State, Brazil [4]. It has since spread throughout many of the important wheat-producing areas of Brazil and to neighboring South American countries including Bolivia and Paraguay. In South America, blast is now a major threat to wheat production [5–7]. Currently, wheat blast affects as much as 3 million hectares, seriously limiting the potential for wheat production in the vast grasslands region of South America.

Blast diseases of grasses are caused by fungal species from the Pyriculariaceae [8] and can occur on 50 grass species [9]. However, a high degree of host specificity exists among and within these fungal species [8, 10]. In South America, wheat blast is caused by isolates of *Magnaporthe oryzae* (syn. *Pyricularia oryzae*) known as pathotype *Triticum* [10–12]. The rice-infecting isolates of *M. oryzae* are genetically distinct from wheat-infecting isolates and generally do not infect wheat [11, 13–20]. Typical symptoms of wheat blast on spikes are premature bleaching of spikelets and entire heads [21–23]. Severely infected wheat heads can be killed, resulting in severe yield losses [21, 22]. The disease is generally spread by infected seeds and airborne spores, and the fungus can survive in infected crop residues and seeds [14]. Little is known about the physiology and genetics of the wheat blast pathogen, and our understanding of the molecular interactions of this pathogen with wheat remains limited.

In February 2016, wheat blast was detected for the first time in Asia with reports of a severe outbreak in Bangladesh relayed through local authorities and the media [24]. Although wheat is not a traditional crop in Bangladesh, its cultivation has expanded in recent years, making it the second major food source after rice [25]. The outbreak is particularly worrisome because wheat blast could spread further to major wheat-producing areas in neighboring South Asian countries, thus threatening food security across the region. Here, we report our immediate response to this plant disease outbreak. To rapidly determine the precise identity and likely origin of the outbreak pathogen, we applied field pathogenomics, in which we performed transcriptome sequencing of symptomatic and asymptomatic leaf samples collected from infected wheat fields in Bangladesh [26, 27]. To promote the project and recruit experts, we immediately released all raw sequence data through a dedicated website Open Wheat Blast (http://www.wheatblast.net). Phylogenomic and population genomic analyses revealed that the Bangladesh wheat blast outbreak was probably caused by isolates belonging to the South American wheat-infecting lineage of *M. oryzae*. We conclude that the wheat blast pathogen was most likely introduced into Asia from South America.

## Results and discussion

### Geographical distribution of the wheat blast outbreak in Bangladesh

**Fig. 1:**
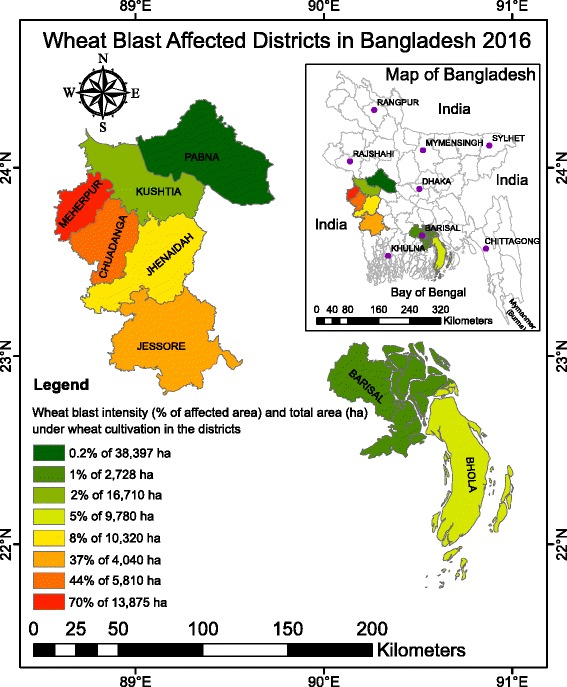
Geographical distribution and severity of the wheat blast outbreak in eight southwestern districts of Bangladesh. The map depicts the intensity of the 2016 wheat blast outbreak across Bangladesh. The percentage of affected area and the total area (hectares) under cultivation are shown for each district based on the color chart

The total area of wheat cultivation in Bangladesh in 2016 was about 498,000 ha (Department of Agricultural Extension, Bangladesh). Wheat blast was observed in eight southwestern districts, viz., Pabna, Kushtia, Meherpur, Chuadanga, Jhenaidah, Jessore, Barisal, and Bhola (Fig. 1). Out of a total 101,660 ha of cultivated wheat in those eight districts, an estimated 15 % were affected by wheat blast.

The severity of wheat blast and associated yield losses varied among districts. The highest percentage of infected wheat fields was observed in Meherpur (70 %) followed by Chuadanga (44 %), Jessore (37 %), Jhenaidah (8 %), Bhola (5 %), Kushtia (2 %), Barisal (1 %), and Pabna (0.2 %) (Fig. 1). Yield losses in different affected districts varied. The highest average yield loss was recorded in Jhenaidah (51 %) followed by Chuadanga (36 %), Meherpur (30 %), Jessore (25 %), Barisal (21 %), Pabna (18 %), Kushtia (10 %), and Bhola (5 %). Although the average yield loss was lower than 51 % across districts, yield losses in individual fields were as high as 100 %. Importantly, 100 % of government-owned Bangladesh Agricultural Development Corporation (BADC) seed multiplication farms in the affected districts (ca. 355 ha) were completely cleared by burning to destroy pathogen inocula by decision of the Ministry of Agriculture (see https://www.youtube.com/watch?v=EmL5YM0kIok). Farmer wheat fields that were severely affected (~100 %) were also burned.

### Wheat blast symptoms in the field

**Fig. 2:**
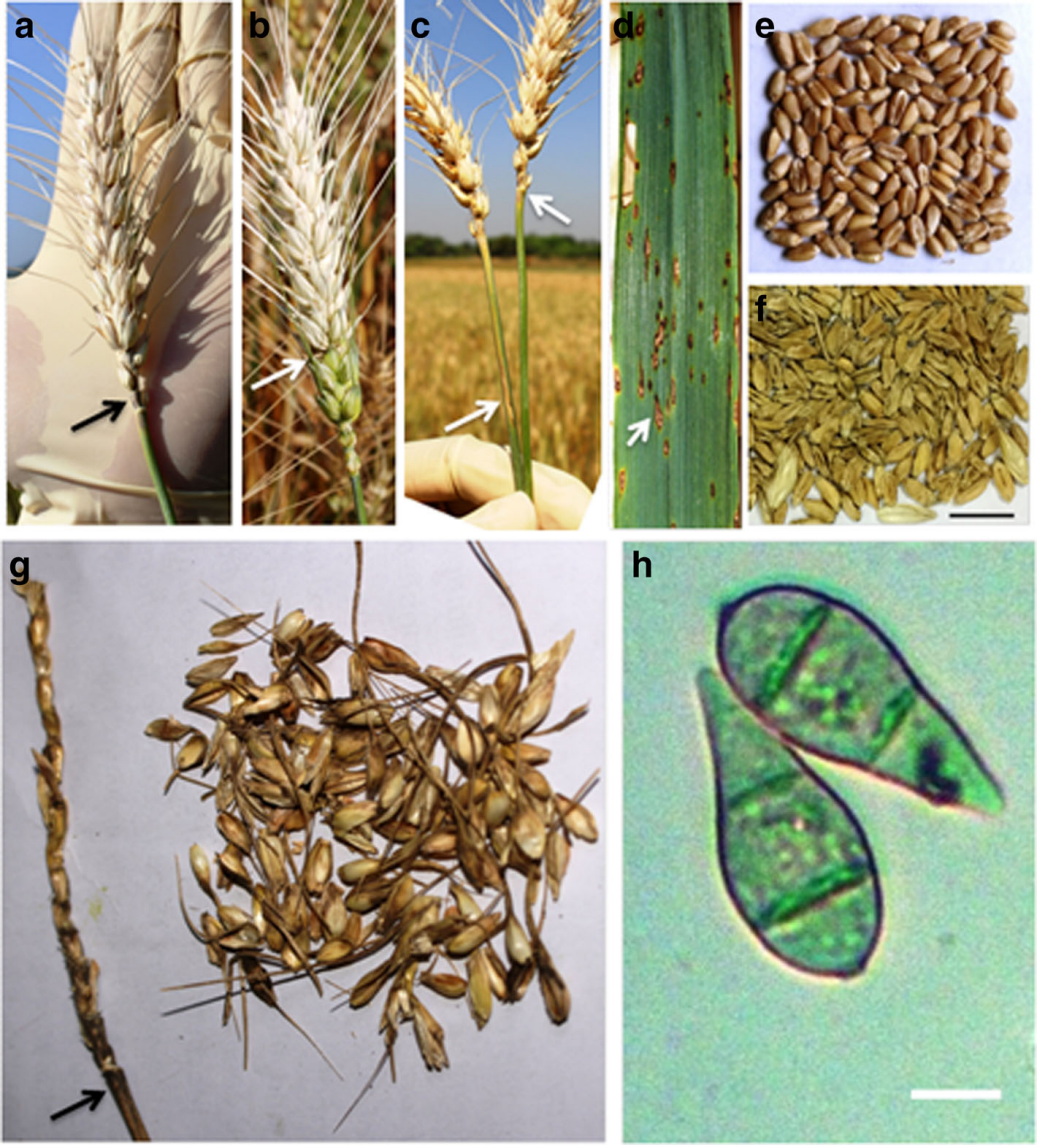
Symptoms of blast disease in spikes, leaves, and seeds of wheat in a farmer’s field in Jhenaidah in Bangladesh, and a micrograph showing two conidia of *Magnaporthe oryzae*. **a** A completely bleached wheat spike with traces of gray from blast sporulation at the neck (*arrow*) of the spike. **b** Complete bleaching of a wheat spike above the point (*arrow*) of infection. **c** Two completely bleached spikes with traces of gray (*upper arrow*) and a lesion (*lower arrow*) from blast sporulation at the base. **d** Typical eye-shaped lesion (*arrow*) and dark gray spots on a severely diseased wheat leaf. **e** Mild blast disease-affected slightly shriveled wheat seeds. **f** Severe blast-affected shriveled and pale wheat seeds. **g** A severely infected rachis with dark gray blast sporulation at the neck (*arrow*) and severely damaged spikelets. **h** Micrograph of two conidia isolated from the infected spike of wheat. Scale bars in **e** and **f** = 1 cm and in **h** = 10 μm

To examine disease symptoms in affected wheat fields, we collected samples from the affected districts. Major symptoms associated with the epidemic included completely or partially bleached (dead) spikes similar to symptoms reported for Brazilian wheat blast epidemics [21, 22] and symptoms reported from Bangladesh in 2016 [23]. The pathogen attacked the base or upper part of the rachis, severely affecting spikelet formation above the point of infection. Complete or partial bleaching of the spike above the point of infection with either no grain or shriveled grain was common in all areas affected by wheat blast (Fig. 2a–c). We commonly observed bleached heads with traces of gray, indicative of fungal sporulation at the point of infection (arrows in Fig. 2a–c and g). In severely infected fields, we also found typical eye-shaped necrotic disease lesions with gray centers in the leaves of some wheat plants (Fig. 2d) [21, 28]. Head infections during the flowering stage resulted in no grain production (Fig. 2g), whereas infection at the grain filling stage resulted in small, shriveled, light in weight, and discolored (pale) grains (Fig. 2e, f) [22].

To determine whether the spike and leaf symptoms on wheat were associated with infection by blast fungi (*Pyricularia* and related genera from the *Pyriculariaceae* sensu; see Klaubauf et al. [8]), we examined infected plant samples using a light microscope. A hallmark of blast fungi is the production of asexual spores that have a specific morphology consisting of three-celled pyriform conidia [8]. Microscopic analyses revealed that gray colored lesions observed on both spikes and leaves carried large numbers of three-celled pyriform conidia from aerial conidiophores (Fig. 2h). This indicates that the fungus present in these lesions belongs to the *Pyriculariaceae*, consistent with a previous report [22]. However, molecular taxonomy tools are needed to determine the species identity.

### Strains isolated from infected wheat samples cause symptoms of wheat blast on artificially inoculated wheat

**Fig. 3:**
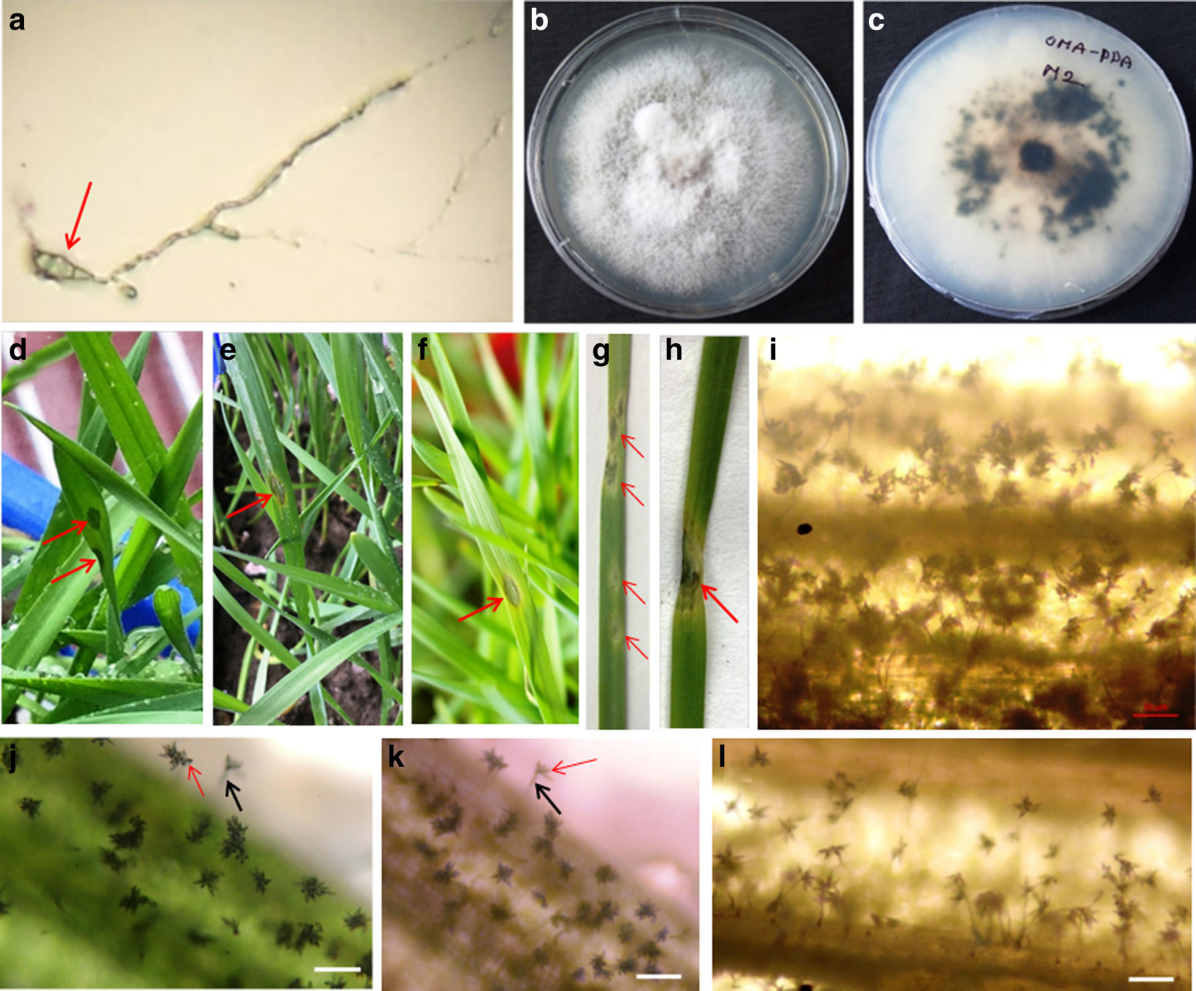
Reinoculation of seedlings with fungal strains isolated from infected wheat seeds. Germinated conidia, growth of mycelia, infection, and sporulation of strains used to artificially inoculate wheat, barley, and goosegrass. **a** A germinated three-celled pyriform conidia (*arrow*) with hyphal growth on water agar medium. **b**, **c** Culture of isolate BTJP 3-1 on PDA plate; upper (*left*) and reverse side (*right*). **d** Photograph showing a diamond-shaped, water-soaked lesion (initial stage of infection symptom, *upper arrow*) on a green wheat seedling leaf five days after conidial inoculation. **e**, **f** Development of an eye-shaped lesion with a gray center (*arrows* in **e** and **f**) on wheat leaves. **g**, **h** A gradual progression of symptoms (*arrows*) on wheat leaves. **i–l** Light micrographs showing massive conidia production (*red arrow*) on aerial conidiophores (*black arrow*) on artificially infected leaves of wheat cultivars Prodip (**i**) and Shatabdi (**j**), goosegrass (**k**), and barley (**l**). Photographs were taken by a camera attached to a microscope at 100× magnification. Scale bars in **j**, **k**, and **l** indicate 50 μm

To confirm whether the fungus found on infected wheat leaves is able to cause the observed symptoms, we isolated ten strains (BTJP 3-1, BTJP 3-2, BTJP 3-3, BTJP 4-1, BTJP 4-2, BTJP 4-3, BTJP 4-4, BTJP 4-5, BTJP 4-6, and BTJP 4-7) using a single-conidia isolation method (Fig. 3a). On potato dextrose agar (PDA) plates, the predominant morphology of the isolates was gray to white aerial mycelia with an olive or brown center (Fig. 3b). After 14–21 days of inoculation, the center of the culture became black (Fig. 3c). Artificial inoculation of wheat seedling leaves using conidia of two isolates (BTJP 3-1 and BTJP 4-1) produced characteristic symptoms five days after inoculation (Fig. 3d–h). Initially, a diamond-shaped, water-soaked lesion in green leaves was observed (Fig. 3d), which gradually turned into an eye-shaped lesion, with a tan or gray colored center (Fig. 3e, f). At a later stage, the spots enlarged, spread to entire leaves, and killed the leaves (Fig. 3g, h). No difference in symptoms was observed on wheat seedlings of the cultivars Shatabdi and Prodip and between the two isolates (BTJP 3-1 and BTJP 4-1). Similar disease symptoms and sporulation were observed on leaves of artificially inoculated goosegrass (*Eleusine indica*) (Fig. 3k) and barley (*Hordeum vulgare*) (Fig. 3l). Terminal infection stages were characterized by a massive production of hyaline to pale gray, pyriform, and asexual conidia on aerial conidiophores. Conidia formation was observed on all infected wheat (*Triticum aestivum*), barley (*H. vulgare*), and goosegrass (*E. indica*) leaves (Fig. 3i–l). Under the same conditions, no visible symptoms or sporulation of conidia were observed microscopically on leaves of artificially inoculated rice (*Oryza sativa* cv. BRRIdhan 49; data not shown). These results are consistent with those of Castroagudin et al. [29] showing that wheat-infecting *M. oryzae* can infect seedlings of barley but is largely asymptomatic on rice. The pathogenicity of wheat blast on *E. indica* is also consistent with reports that *E. indica* is a major alternate host in South America [30, 31]. *E. indica* is also a common weed in the highlands of Bangladesh and may similarly serve as a alternate host of wheat blast. Understanding the role of alternate hosts in disease cycles and epidemics of wheat blast will be key in formulating effective disease management strategies.

### Transcriptome sequencing of wheat leaf samples from Bangladeshi fields

**Fig. 4:**
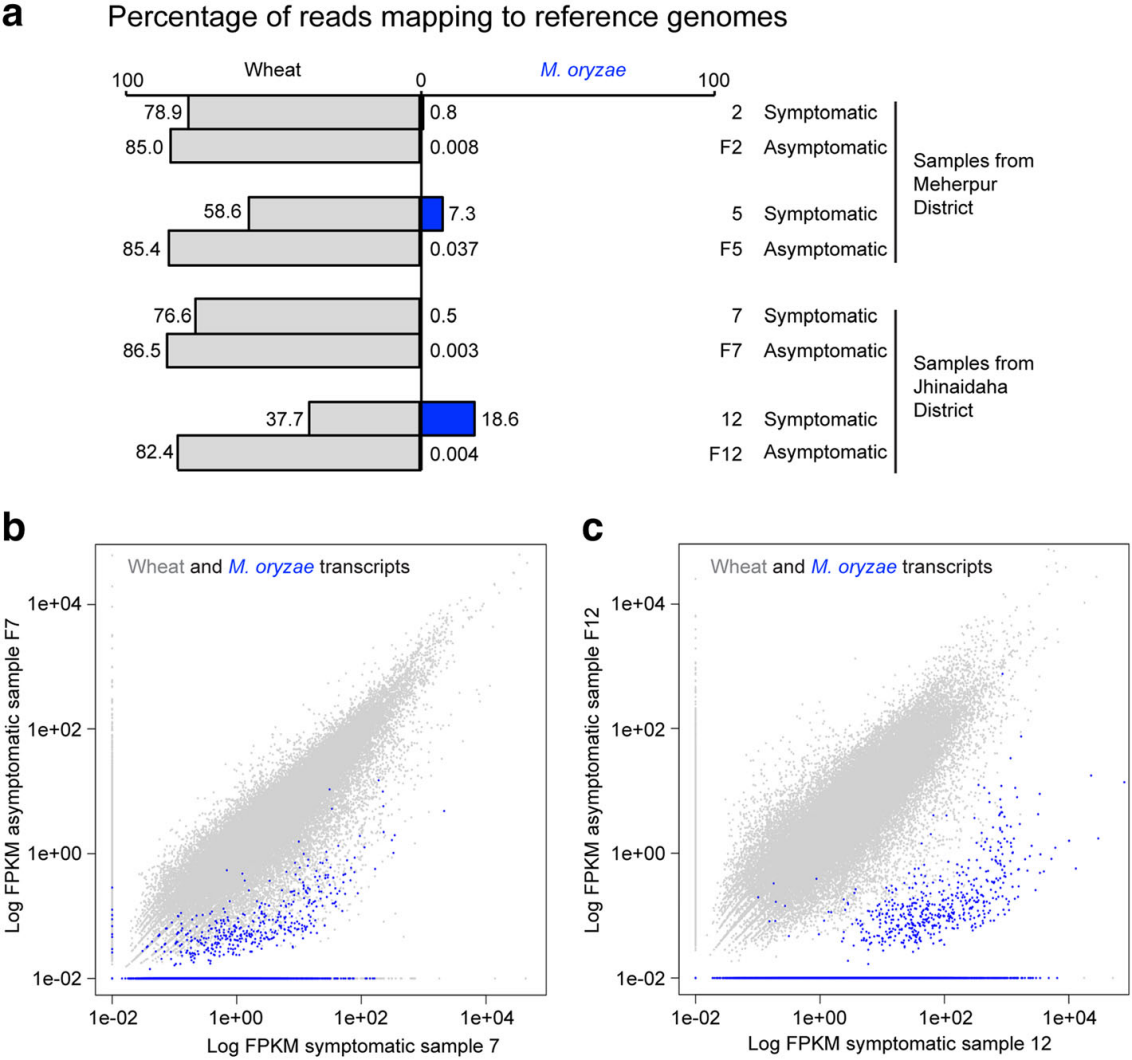
Transcriptome sequencing of infected leaves from farmer fields reveals *Magnaporthe oryzae* transcripts in symptomatic samples. **a** Comparison of sequence read mapping data from the four sample pairs to the genomes of wheat blast fungus *M. oryzae* BR32 (in *blue*) and wheat (*light gray*). **b**, **c** Scatter plots of fragments per kilobase of transcript per million (*FPKM*) values from sample pair 7-F7 (**b**) and 12-F12 (**c**) aligned to the combined transcriptomes of wheat and *M. oryzae* BR32. Transcripts from wheat (100,344) are shown in *light gray* and transcripts from *M. oryzae* BR32 (14,349) are shown in *blue*

We used field pathogenomics [26] to identify which blast fungus species was present in infected wheat fields in Bangladesh. We collected samples of both symptomatic and asymptomatic leaves from wheat fields in different regions of Bangladesh, including Meherpur and Jhenaidah districts, and extracted total RNA from four pairs of symptomatic (samples 2, 5, 7, and 12) and asymptomatic samples (samples F2, F5, F7, and F12) (Additional file 1: Table S1). We prepared and sequenced RNA-seq libraries using Illumina technology, yielding 68.8 to 125.8 million 101-bp pair-end reads with an average insert size of 419 bp. Next, following data trimming, we aligned high-quality reads to both the *M. oryzae* wheat blast fungus BR32 and wheat genomes [19, 32]. Sequence reads from all samples with disease symptoms aligned to the BR32 genome, ranging from 0.5–18.6 % of the total reads (Fig. 4a). By contrast, only a minor proportion of the reads from the asymptomatic samples aligned to the BR32 genome (range: 0.003–0.037 %, Fig. 4a–c). Between 37.7 % and 86.5 % of total reads aligned to the wheat genome sequence (Fig. 4a). We obtained similar numbers when considering the reads aligning to *M. oryzae* and wheat transcriptomes (Additional file 2: Table S2). Variation in percentage mapped reads of host and fungal transcripts among symptomatic samples is most likely explained by differences in the disease severity and infection stage among field collected leaves. The finding that on average 6.8 % reads per sampled transcriptome aligned to the wheat blast genome BR32 indicated that *M. oryzae* is present in symptomatic (infected) wheat samples from Bangladesh.

### Bangladesh wheat blast outbreak was likely caused by a wheat-infecting South American lineage of *M. oryzae*

**Fig. 5:**
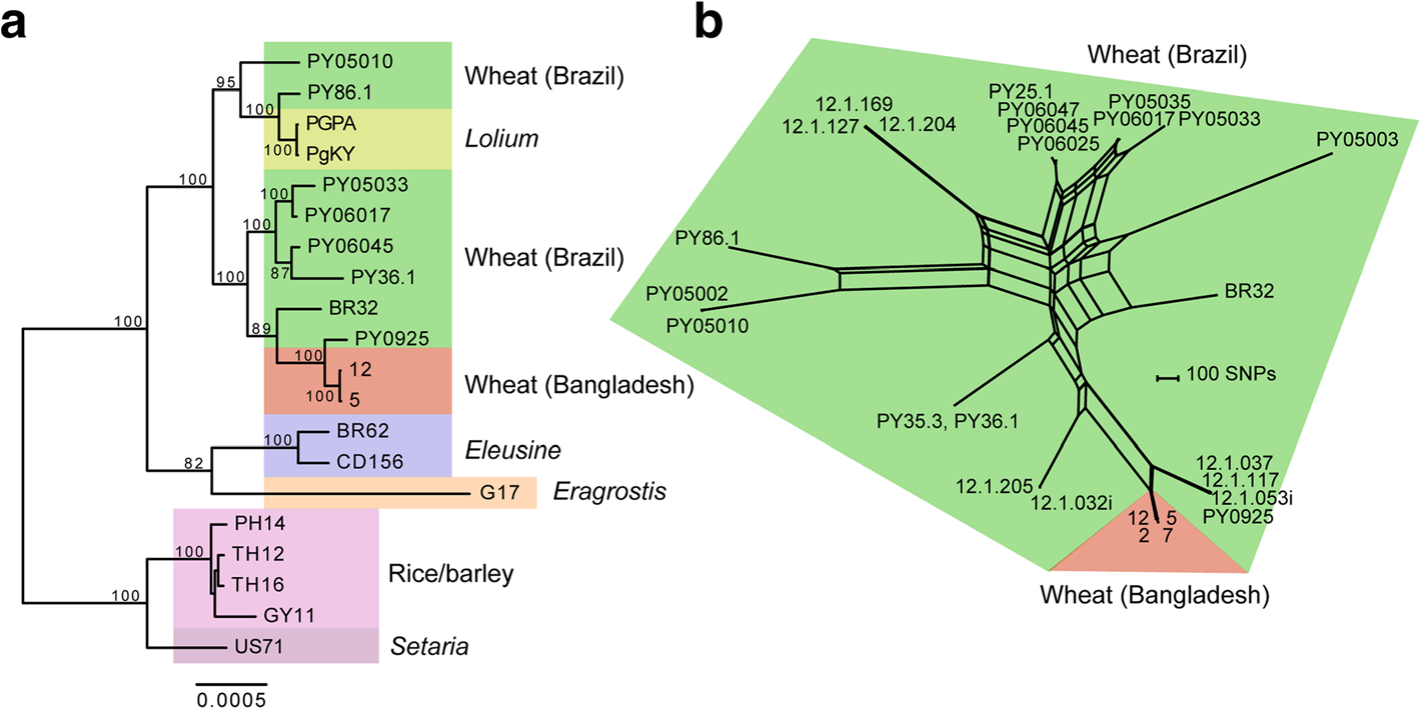
The origin of the Bangladesh wheat blast fungus. **a** Maximum likelihood genealogy inferred from the concatenation of aligned genomic data at 2193 orthologous groups of predicted transcript sequences. Scale bar represents the mean number of nucleotide substitutions per site. **b** Population genomic analyses of transcriptomic single nucleotide polymorphisms among *M. oryzae* isolates from wheat in Brazil and Bangladesh. The network was constructed using the Neighbor-Net algorithm. The scale shows the number of informative sites

We used phylogenomic approaches to determine how related the fungal pathogen detected in wheat leaf samples from Bangladesh is to *M. oryzae* lineages infecting cereals and grasses. We also performed population genomics analyses to gain insight into the geographic origin of these Bangladeshi isolates using a set of sequences from wheat-infecting *M. oryzae* isolates collected in Brazil over the last 25 years. We first determined the taxonomic affiliation and phylogenetic position of wheat-infecting Bangladeshi samples. To this aim, we extracted predicted transcript sequences from the assembled genomic sequences of 20 *M. oryzae* strains isolated from infected rice (*O. sativa*), wheat (*T. aestivum*), foxtail millet (*Setaria* spp.), *Eleusine* spp., *Lolium* spp., and *Eragrostis* spp. [19] (this study; see Additional file 1: Table S1 for full details). We identified 2193 groups of sequences with orthologous relationships across the 20 reference transcriptomes and the two Bangladeshi isolates that had the largest number of genes represented in their transcriptomic sequences. We aligned orthologous transcripts, processed alignments, and inferred a maximum likelihood genealogy based on the concatenated sequences using RAxML [33]. The Bangladeshi isolates clustered with high bootstrap support (>90 %) with wheat-infecting isolates of *M. oryzae* (Fig. 5a), indicating that the emergence of wheat blast in Bangladesh was caused by isolates belonging to the known *M. oryzae* wheat-infecting lineage, and not by an unknown Pyriculariaceae species or a novel *M. oryzae* lineage.

Given that the Bangladesh outbreak was caused by isolates related to known wheat-infecting lineages of *M. oryzae*, our next step was to infer genealogical relationships between Bangladeshi and South American wheat blast samples. We performed population genomics analyses using transcriptomic single nucleotide polymorphisms (SNPs) identified by aligning sequence reads to the *M. oryzae* reference genome 70-15 [34]. We included all four symptomatic samples from Bangladesh and a diverse collection of 23 *M. oryzae* wheat-infecting isolates sampled from Brazil, the main wheat growing country affected by wheat blast (Additional file 1: Table S1). As the wheat blast isolates from Brazil were sequenced from genomic DNA, we restricted the analyses to transcriptomic SNPs genotyped at high confidence in the symptomatic Bangladeshi sample 12, retaining a total of 15,871 SNPs. Since the reproductive mode of wheat blast populations can be mixed, including both sexual and asexual reproduction [18], we chose to build a Neighbor-Net network that takes into account potential recombination among genotypes. The network analyses identified small groups of near-clonal genotypes (e.g., isolates 12.1.205 and 12.1.032i), whereas all other isolates appeared genetically distinct and displayed reticulate evolution. The Bangladesh outbreak isolates grouped as a near-clonal genotype that was most closely related to a group of Brazilian wheat-infecting isolates from Minas Gerais, São Paulo, Brasília, and Goiás (strains PY0925, 12.1.053i, 12.1.117, and 12.1.037, respectively). Systematic analyses of recent wheat-infecting isolates from Brazil and neighboring countries will be needed to ascertain the most likely infection route from South America. Also, additional phylogenomic work, based on deeper sampling of the diversity of grass-infecting *M. oryzae*, will provide further insight into the genealogical relationships among host-specific lineages and the timing of lineage splitting/merging events.

## Conclusion

Our rapid open source genomic surveillance approach has revealed the precise identity of the infectious Bangladeshi fungus as the known wheat-infecting *M. oryzae* lineage and indicated that it most likely originated from South America. This finding calls for intensive monitoring and surveillance of the wheat blast pathogen to limit its further spread outside South America and Bangladesh. In addition, our finding indicates that the knowledge acquired to manage wheat blast in Brazil using disease resistant cultivars [35–37] and fungicides [38, 39] can be directly applied to the Bangladeshi epidemic.

## Methods

### Field data

The dates of first incidence of disease and areas of wheat cultivation and blast-infected fields in different districts of Bangladesh were obtained from the Department of Agricultural Extension (DAE) of Bangladesh. To verify the data obtained by the DAE on the severity of the wheat blast epidemic, a second data set on yield loss was directly collected from the farmers (*n* = 100) of the most severely infected wheat blast district, Meherpur, through face-to-face interviews of randomly selected farmers after harvesting the crop. Among 15,471 ha with wheat blast in Bangladesh, the disease incidence in the Meherpur district alone involved 9640 ha, approximately 62 % of the total wheat blast area in the country.

### Isolation of wheat blast strains from infected seeds and inoculation of wheat seedlings

Fungal strains were isolated from infected and shriveled wheat seeds collected from the farmers of the Jhenaidah district of Bangladesh. Infected wheat seeds were surface sterilized successively with 95 % ethanol followed by 10 % sodium hypochlorite and 95 % ethanol [28]. The seeds were kept in a Petri dish laid with sterilized filter paper maintaining abundant moisture. Samples were checked every day under the microscope to monitor the production of conidia and conidiophores on wheat seeds. After 3 days of incubation at room temperature (ca. 30 °C), abundant conidia on aerial conidiophores were observed. Seeds with conidia were transferred to an Eppendorf tube containing 1 ml of sterilized water and vortexed for 1 min at 700 rpm. A conidial suspension was separated from the seed, diluted 100-fold with sterilized water, and then spread on 1.5 % water agar medium and incubated for 2 days [40]. Each plate was observed under a microscope at 100× magnification to identify germinated single conidia with hyphal growth. Germinated conidia were transferred on an agar block and placed on a PDA plate for 7 days of incubation. Repeated cultures were established from the tip of growing hyphae for further purification. For the production of conidia, hyphal blocks from a fully grown plate were transferred into water agar containing sterilized wheat leaves 40 g L^−1^, streptomycin 50 mg L^−1^, tetracycline 50 mg L^−1^, and chloramphenicol 50 mg L^−1^ [28]. Conidia were harvested from 14-day-old culture plates flooded with sterilized water containing 0.01 % Tween 20 and gently scraped with an inoculation loop to dislodge conidia from conidiophores [41]. The conidial suspension was then filtered through two layers of cheesecloth and adjusted to 5 × 10^3^ conidia per ml. Seedlings of two wheat varieties, Prodip and Shatabdi, were grown from surface sterilized seeds in autoclaved soils and in plastic trays. Seedlings were sprayed with a conidial suspension of two purified isolates, BTJP 3-1 and BTJP 4-1, until full wet. Non-inoculated controls were sprayed with a solution of sterilized water and Tween 20. Inoculated plants were immediately covered with sterilized transparent polyethylene bags to maintain humidity. Plants were kept under natural light conditions at 30–32 °C for the development of blast symptoms. For the production of conidia on aerial conidiophores on symptomatic wheat leaves, excised plant leaves were placed on wet filter paper in a Petri dish. Sterilized pipette tips were used to support the diseased tissues so that they were in contact with wet filter paper to prevent desiccation. The Petri dish was covered with a lid and placed at room temperature (30–32 °C). After incubation for 24 h, infected leaves were examined under a light microscope to confirm sporulation and then photographed. Strains were reisolated and preserved on dried filter paper at 4 °C for further examination. Seedlings of barley (*H. vulgare*), rice (*O. sativa*), and goosegrass (*E. indica*) were also inoculated by conidia produced by the strains BTJP 3-1 and BTJP 4-1 following the protocols described for wheat seedlings.

### Transcriptome sequencing of field collected samples

Leaf blades from wheat displaying blast symptoms and those with no symptoms were harvested from the same fields, cut into thin strips (approximately 0.5 × 1.0 cm), and immediately stored in 1 ml RNAlater solution (Thermofisher Scientific, Basingstoke, UK). Total RNA was extracted from the samples using the RNeasy Plant Mini kit (Qiagen, Manchester, UK) following the manufacturer’s instructions. The amount and the quality of RNA samples were determined using the Agilent 2100 Bioanalyzer (Agilent Technologies, Edinburgh, UK). cDNA libraries were prepared using the Illumina TruSeq RNA Sample Preparation Kit (Illumina, Cambridge, UK). Library quality was confirmed before sequencing using the Agilent 2100 Bioanalyzer (Agilent Technologies, Edinburgh, UK). The libraries were sequenced on the Illumina HiSeq 2500 system (Illumina) operated by The Genome Analysis Centre, UK, producing 101-bp paired-end reads. The reads were mapped to the genomes of wheat and wheat blast strain *M. oryzae* BR32 using the TopHat software, version 2.0.11 [42], and fragments per kilobase of transcript per million (FPKM) values of mapped reads to the transcriptomes were calculated using Cufflinks, version 2.1.1 [43]. De novo assembly of transcriptomes was performed using sequence reads from each sample with Trinity software, version 2.06 [44]. Within days of sequencing, the data were made public on Open Wheat Blast (http://www.wheatblast.net). A timeline from sample collection to population and phylogenomic analysis is provided on the Open Wheat Blast website (http://s620715531.websitehome.co.uk/owb/?p=485).

### Population and phylogenomic analyses

We used predicted transcript sequences extracted from the assembled genomic sequences of 20 *M. oryzae* isolates collected on infected leaves of rice (*O. sativa*), wheat (*T. aestivum)*, foxtail millets (*Setaria* spp.), *Eleusine* spp., *Lolium* spp., and *Eragrostis* spp. (Additional file 1: Table S1) [18, 19, 45–47]. We used Proteinortho [48] to identify groups of sequences with orthologous relationships across the 20 reference transcriptomes and each of the Bangladeshi transcriptomes. We identified 983, 3250, 501, and 3413 groups of orthologous sequences across the reference transcriptomes from samples 2, 5, 7, and 12, respectively. Only the two Bangladeshi isolates that had the largest number of orthologous sequences were retained for further analysis (samples 5 and 12). The consensus set of orthologous transcripts across the 22 transcriptomes included 2193 groups of sequences. We aligned orthologous groups of sequences using MACSE, with default parameters [49].

We removed codons with missing data or alignment gaps. We excluded transcript alignments for which >0.5 % of sites corresponded to singletons or doubletons exclusive to the Bangladeshi isolates, suggesting erroneous assignment of predicted sequences to *M. oryzae* BR32 transcripts or sequencing errors in transcript assemblies. We also excluded the regions corresponding to the first 30 and last 16 codons and treated ambiguities as missing data. Maximum likelihood phylogenetic inference was performed on the concatenated sequence of 1923 orthologs (2,676,792 bp in total), using the GTRGAMMA model in RAxML version 8.1.17 with 100 bootstrap replicates [33]. The maximum likelihood genealogy was mid-point rooted along the longest branch, which was the branch connecting the foxtail millet- and rice-infecting lineages to other lineages.

For population genomic analyses, we identified transcriptomic SNPs based on short read alignments against the *M. oryzae* reference genome 70-15. We mapped quality-trimmed Illumina short read data generated from RNA using TopHat version 2.0.14 [43]. For all completely sequenced genomes, we aligned quality-trimmed Illumina short read data against the reference genome 70-15 using Bowtie version 2.2.6 [50]. For all strains collected from the Bangladesh outbreak, transcriptomic sequences were aligned using TopHat version 2.0.14. We identified variants in the genomes of the different strains using the Genome Analysis Toolkit (GATK) version 3.5 from the Broad Institute [51]. We used a two-step variant calling according to the GATK best practice guidelines. We first called raw variants with local reassembly of read data using Haplotype caller. All raw variant calls were jointly genotyped using GenotypeGVCF. We used SelectVariants to subset the variant calls to contain only SNPs. Then, the SNPs were hard-filtered using the following criteria: QUAL ≥ 5000.0, QD ≥ 5.0, MQ ≥ 20.0, –2.0 ≤ ReadPosRankSum ≤ 2.0, –2.0 ≤ MQRankSum_upper ≤ 2.0, –2.0 ≤ BaseQRankSum ≤ 2.0. Furthermore, we only retained SNPs genotyped in at least 90 % of all strains and genotyped the Bangladeshi sample 12 (Additional file 1: Table S1). We used SplitsTree version 4.14.2 to generate a Neighbor-Net network from Brazilian and Bangladeshi wheat blast strains [52]. To build the network, we used uncorrected *p* distances calculated from the SNP supermatrix. The network was drawn based on equal angle splits.

## Additional files

Additional file 1:
**Table S1.** Samples included in the phylogenomic and population genomic analyses. (XLSX 15 kb) Additional file 2:
**Table S2.** Short read coverage of *Magnaporthe oryzae* and wheat transcriptomes in Bangladeshi samples. (PDF 66 kb)
