# Supplementary material for: Emergence of wheat blast in Bangladesh was caused by a South American lineage of Magnaporthe oryzae
Source: BMC Biol. 2016 Oct 3;14:84. doi: 10.1186/s12915-016-0309-7 (PMC5047043; doi:10.1186/s12915-016-0309-7)
Supplement: Additional file 2: — Table S2. Short read coverage of Magnaporthe oryzae and wheat transcriptomes in Bangladeshi samples. (PDF 66 kb) [file 12915_2016_309_MOESM2_ESM.pdf]

Table S2. Short read coverage of *Magnaporthe oryzae* and wheat transcriptomes in Bangladeshi samples

Sample Name	No. of *M. oryzae* transcripts with FPKM > 0.5[a,b]	% of *M. oryzae* transcriptome (14349 transcripts)[b]	No. of wheat transcripts with FPKM > 0.5[a,c]	% of wheat transcriptome (100344 transcripts)[c]
12 (symptomatic)	9604	66.93	52245	52.07
7 (symptomatic)	2661	18.54	53850	53.67
F12 (asymptomic)	88	0.61	54914	54.73
F7 (asymptomatic)	31	0.22	52962	52.78

[a]Illumina short reads were mapped to the transcriptomes using bowtie2 (v2.1.0) and FPKM values were calculated with cufflinks (v2.1.1).
[b]Transcriptome of *M. oryzae* BR32 downloaded from http://genome.jouy.inra.fr/gemo/ was used as a reference.
[c]Transcriptome of wheat downloaded from http://plants.ensembl.org/Triticum_aestivum/ was used as a reference.
